# Perinatal mortality and its predictors in Beni City, Democratic Republic of Congo: a cross-sectional study

**DOI:** 10.1186/s40748-024-00184-6

**Published:** 2024-07-05

**Authors:** Mathe Julien Kahiririaa, Josephine Namyalo, Nasur Mubarak, Emmanuel Otieno

**Affiliations:** 1 Department of Public Health, Uganda Christian University, Mukono, Uganda; 2 Department of Public Health, Official University of Ruwenzori, Butembo City, Democratic Republic of Congo; 3 Yumbe Regional Referral Hospital, Ministry of Health, Yumbe, Uganda; 4 School of Public Health, Gudie University Project, Kampala, Uganda; 5 Uganda Peoples Defense Forces, Kampala, Uganda

**Keywords:** Perinatal mortality, Apgar score, Birth weight, Democratic Republic of Congo

## Abstract

**Background:**

Globally, perinatal mortality rates have decreased considerably in the last 30 years. However, in sub-Saharan African countries perinatal mortality remains a public health burden. Therefore, this study aimed to determine the Perinatal Mortality Rate and the factors associated with perinatal mortality in Beni City, Northeastern Democratic Republic of Congo.

**Methods:**

A hospital-based retrospective cross-sectional study was conducted among 1394 deliveries that were documented in Beni General Referral Hospital from 2 January to May 31, 2022. The study was done in the conflict-ridden Beni city of the North Kivu Province. Analysis was done using Open Epi and SPSS version 22. Binary and Multivariate logistic regression analyses were performed. Odds ratio with 95% confidence interval was used to measure strength of association.

**Results:**

Findings indicate that 60.7% of 1394 participants were below the age of 21 years, and 95.1% (1325) Beni residents. The Perinatal Mortality Rate was 42.3 per 1000 live births. Majority (51) of the postpartum women who experienced perinatal mortality didn`t have a history of perinatal mortality as compared to their counterparts. Multivariable analysis revealed that birth weight (AoR = 0.082, 95% CI 0.014–0.449, *p* < 0.05) and Apgar score in the 10th minute (AoR = 0.082, 95% CI 0.000- 0.043, *p* < 0.05) were significantly associated with Perinatal mortality.

**Conclusion:**

The high perinatal mortality rate in Beni General Referral Hospital, approximately four in every 100 births remains a disturbing public health concern of which is attributable to low birth weight and Apgar score. This study may help policy-makers and healthcare providers to design preventive interventions.

## Background

Being a mother is a desire that most women aspire to at some point in their lives. However, a risk of perinatal death persists as a global public health concern, particularly in lower-middle-income countries (LIMCs) such as Democratic Republic of Congo (DRC). Perinatal mortality is an indicator of the quality of health that echoes poor healthcare in each society, socio-economic status of a country [1]. Perinatal mortality is defined as stillbirths (mortality of fetuses aged 28 weeks of gestation) and early neonatal mortality (mortality within 7 days after birth). Globally, over five million perinatal deaths occur annually contributing to 4% of the global disease burden. The “Every Newborn Action Plan (ENAP)” platform was launched in 2014 to decline perinatal mortality to less than 10 per 1,000 total births of countries by 2035 [2, 3]. The sub-Saharan Africa (SSA) which is home to 16% of the World’s total population shoulders the highest perinatal mortality at 34.7 to 42.95 mortality per 1000 births [4]. In addition, the risk of a woman experiencing a stillbirth in SSA of 21.0 per 1,000 births is seven times more likely than the lowest rate of 2.9 in Europe, North America Australia, and New Zealand [5]. The DRC still suffers neonatal mortality rate and still birth rates at 27 per 1000 births respectively and one of the highest PMR in the World at 40 per 1000 live births [5, 6]. Nearly, this is three times the UN Sustainable Development Goal (SDG) and Every Newborn Action Plan (ENAP) target of ≤ 12 deaths per 1000 livebirths by 2030 [7]. However, this is a marked decrease from the previous 77 per 1,000 births in 2006 [8]. Despite increased survival rates due to improved perinatal medicine, low universal health coverage, low meagre allocations at 8.5% of the budget and 3.5% GDP, poor adherence to 2016 WHO ANC and 2013 Post Natal Care guidelines still contribute to mortality [9–11].

Studies conducted in various hospitals in Eastern DRC pitted by war and decades of conflict indicated perinatal mortality rate was 235 per 1000 births in Lomami Province, 32 per 1000 at Dr. Rau-Ciriri Hospital, in Bukavu and 27 per 1000 for the town of Lubumbashi respectively [12, 13].

There is paucity of data on perinatal deaths in DRC in spite the few studies that have been done. Additionally, the recent Demographic Health Survey 2013–2014 does not mention about perinatal health. Yet, DRC is second in Africa with the highest neonatal mortality rate [14]. Moreover, previous studies show factors associated with perinatal deaths were identified as, maternal associated diseases, fetal hypotrophy, advanced maternal age, prematurity, and insufficient antenatal care. The challenge is the anecdotal evidence which suggests that Beni has poor perinatal outcomes and probably high PMR. Therefore, this study aimed to determine the perinatal mortality rate and the factors associated with perinatal mortality in Beni General Referral Hospital, Democratic Republic of Congo which will contribute evidence-based data in the decrease of the furthermost preventive tragedy and mortality indicator today.

## Methods

### Study design

We conducted this retrospective hospital facility-level cross-sectional in Beni Hospital which is a public tertiary care referral hospital in Democratic Republic of Congo, from 2 January to May 31, 2022. The study was done among mothers who delivered babies.

### Study setting

The study was conducted at Beni General Referral Hospital located in the conflict-ridden Beni city of the North Kivu Province (NKP) Northeastern DRC. Beni is at a crossroads of population movements from Congolese, Ugandan, Rwandan and, to a lesser extent, Burundian territories. This area is experiencing extensive horrific violence in the country since more than two decades [15, 16].

### Study variables

The main outcome was perinatal mortality. The explanatory variables were Apgar score, birth weight, foetal heartbeat, foetal presentation, age birth, sex, maternal age, maternal residence, maternal occupation, type of referral, Hospital catchment area, birth space, history of perinatal mortality, type of pregnancy, parity, and delivery method. The explanatory variables were selected based on recommended perinatal indicators [3]. Perinatal Mortality Rate (PMR) was used to determine prevalence of perinatal mortality. We defined PMR as a ratio of still births and early neonatal mortality expressed per 1000 births [17]. It was measured nominally as 1 = Yes (still births or early neonatal mortality), and 2 = No question (Baby survived during the perinatal period. Stillbirth was defined as a baby born with no signs of life recognized to have died after 24 weeks of gestation [18].

### Study participants and eligibility criteria

We included all babies born alive or born dead aged at least 28 weeks of gestation within the study period; and babies who died or survived within the first week of life during the study period were included in the study. Babies aged less than 28 weeks of gestation or unknown gestational age at birth; and babies who died after 7 days of life, and babies born before or after the study period were excluded. Also, all babies and mothers with incomplete data and major congenital anomalies were excluded from the study.

### Sampling procedure and sample size determination

The sample size was 1394 based on previous similar study [19]. The study population comprises all deliveries after 28 weeks of gestation in Beni GRH over a five-month period. This was because of a high increasing of delivery cases in Beni Hospital from January to May 2022, because an International NGO (MSF) engaged to pay the fees of maternity care, and the records were well arranged. We selected purposively Beni General Referral Hospital because it provides maternal and neo-natal care geographically in North Kivu Province a region with the largest proportion of maternal deliveries at 80% of the 92% occur in government-owned facilities. The consecutive sampling technique was used to select babies and mothers based on inclusion criteria and available records.

### Data collection tools and procedure

Maternal and baby data were collected from the hospital records. The key data source was the partographs. Others included labor ward delivery register, the neonatology unit register, the laboratory result sheet, the reference note, and the operatory protocol sheet. The questionnaire had three sections: characteristics of mother, characteristics of the baby, obstetrical characteristics of mothers and Obstetrical characteristics of postpartum women.

### Quality control

A questionnaire was developed specifically for this study based on the objectives of the study and used for data collection from mothers and babies who met the inclusion criteria of the study. The questionnaire was refined by the Authors with reference WHO, UNFPA, and UNICEF modules used to capture key indicators of availability, use, and quality of Emergency Obstetric and Natal Care services. The information written in French language in the records were translated in English. The survey was written in English and translated to Kiswahili and Kinande, and back translated to account for culturally sensitive wording and reviewed for content validity. Although French is the lingua franca of DRC, the area of the survey is largely Kiswahili and Kinande speaking. The pretest was done prior to the study for ease of understanding and consistency of tool. Three research assistants all registered nurses were recruited and received 3-day training for data collection.

### Ethical consideration

The Ethics Committee of the school of research and postgraduate studies of Uganda Christian University approved the study. Authorization for accessing the hospital services was obtained from the Manager Administrator of the Health Zone, and the Administrator Manager of the Beni General Referral Hospital. Informed consent forms of all participants were obtained. The study was conducted in accordance with the Declaration of Helsinki (as revised in 2013). The information collected was treated as confidential and codes were used to identify participants and not names.

### Data processing and analysis

Data collected through the questionnaires were keyed in using Open Epi and exported to SPSS, version 22.0 for data analysis. Frequencies and proportions were for categorical variables. Binary and Multivariate logistic regression analyses were performed to assess the factors associated with perinatal mortality. Odds ratio with 95% confidence interval was used to measure strength of association. The level of statistical significance was defined as *p* < 0.05.

## Results

### Characteristics of mothers and babies

Findings show 60.7% (408) of mothers were below the age of 21 years, 58.4% (814) unemployed., most mothers were from the hospital catchment area Beni at 95.1% (1325), majority 33.4% (466) being residents of Beni 54.3% (*n* = 757) of the mothers arrived at the hospital without being referred while 45.7% (637) were referred to the hospital. Regarding neonate characteristics 80.6% (1124) weighed between 2500 and 4000 g at birth and 95.3% (1329) of the fetus had a normal heartbeat between 110 and 160 bpm (Table 1). The study revealed PMR was 42.3% (Approximately 42 per 1000 births) i.e., out of 1394 births, 95.77% (957.7 per 1000 births) survived during the perinatal period. Table 1 demonstrates the least postpartum women having experienced perinatal mortality were 4.7% (*n* = 65) whereas 54.7% (*n* = 762) were the majority having given birth 1–4 times. Most 42.6% (594) postpartum women gave birth in space of 2 to 4 years, the highest number of them giving birth to singleton about 94.8% (1321) and 50.1% (699) of the postpartum women gave birth from the SVD method of delivery.

**Table 1:** Characteristics of mothers and neonates,2022

Variables	Frequency	Percent
Maternal characteristics		
Age (years)		
<21	408	60.7
21–25	846	29.3
>35	140	10.0
Occupation		
Employed	39	2.8
Unemployed	814	58.4
Personal Business	209	15.0
Agriculture	332	23.8
Commune of Residence		
Ruwenzori	110	7.9
Beu	466	33.4
Bungulu	416	29.8
Mulekera	336	24.1
Out of Beni Town	66	4.7
Hospital catchment area		
Beni	1325	95.1
Other than Beni	69	4.9
Type of referral		
Self-Reference	757	54.3
Referred	637	45.7
Neonate characteristics		
Sex		
Male	724	51.9
Female	670	48.1
Birth Weight (Grams)		
1000–1499	19	1.4
1500–2499	244	17.5
2500–4000	1124	80.6
>4000	7	0.5
Age Birth Category		
Preterm birth	58	4.2
Term birth	1331	95.5
Post term birth	5	0.4
Fetal heartbeat (beats per minute: bpm)		
Not perceived	44	3.2
110–160	1329	95.3
<110/ >160	21	1.5
Fetal Presentation		
Head	1337	95.9
Malpresentation	57	4.1
Obstetrical characteristics of postpartum women		
Birth space (years)		
<1	369	26.5
1 < 2	256	18.4
2–4	594	42.6
>4	175	12.6
History of Perinatal Mortality		
Yes	65	4.7
No	1329	95.3
Method of delivery		
SVD	699	50.1
AVD	3	0.2
Cesarean section	692	49.6
Type of Pregnancy		
Singleton	1321	94.8
Twins	73	5.2
Parity		
1–4	762	54.7
More than 4	252	18.1
Never gave birth	380	27.3

### Babies apgar score

Majority 87.0%, 91.5%, 95.7% of the babies had an Apgar score of 8 to 10 in the 1st, 5th and 10th minute respectively while minority 5.3%, 3.4% had an Apgar score of 0 to 4 in the 1st and 5th minute respectively. In the 10th minute, minority 1.0% of the babies had an Apgar score of 5 to 7.

**Fig. 1:**
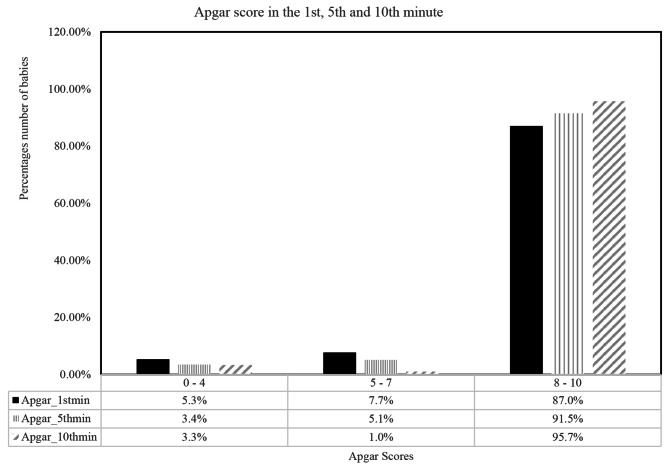
Babies apgar scores

### Perinatal mortality between maternal and neonate characteristics

Women`s commune of residence was statistically associated with perinatal mortality (0.018, *p* ≤ 0.05). Also, majority 49.2% (29) of the women who experience perinatal mortality being residents of Beni. Regarding the neonates, the birth weight, age birth category, fetal heartbeats, foetal presentation, Apgar Score in the 1st, 5th and 10th minute were all statistically significant at *p* ≤ 0.05 on running a Chi2 test on these variables against Perinatal Mortality. Hence considered to be likely contributors to perinatal mortality. Perinatal mortality was high among babies who had a foetal head presentation and Apgar scores of between 0 and 4 in the 1st minute at 52 (Table 2).

**Table 2:** Bivariate analysis for perinatal mortality between maternal and neonate characteristics,2022

Variables	Perinatal Mortality		*P*- Value
	Yes	No	
Age (years)			0.651
<21	17	391	
21–25	34	812	
>35	8	132	
Occupation			0.907
Employed	2	37	
Unemployed	36	778	
Personal Business	7	202	
Agriculture	14	318	
Commune of Residence			0.018
Ruwenzori	1	109	
Beni	29	437	
Bungulu	11	405	
Mulekera	17	319	
Out of Beni Town	1	65	
Hospital catchment area			0.572
Beni	57	1268	
Other than Beni	2	67	
Type of referral			0.178
Self-Reference	27	730	
Referred	32	605	

### Perinatal mortality and postpartum women

It`s observed that history of perinatal mortality and type of pregnancy were statistically significant when related with perinatal mortality (*p* = 0.001, ≤ 0.05), implying that there is a statistical relationship between history of perinatal mortality and type of pregnancy with perinatal mortality (Table 3). Majority (51) of the postpartum women who experienced perinatal mortality didn`t have a history of perinatal mortality as compared to their 8 counterparts.

**Table 3:** Bivariate analysis between perinatal mortality and postpartum, women 2022

Variables	Perinatal Mortality		*P*- Value
	Yes	No	
Maternal characteristics			
Birth space (years)			0.247
<1	17	352	
1 < 2	9	247	
2–4	21	573	
>4	12	163	
History of Perinatal Mortality			0.001^*^
Yes	8		
No	51	1278	
Method of delivery			0.730
SVD	27	672	
AVD	0	3	
Cesarean section	32	660	
Type of Pregnancy			0.000
Singleton	49	1272	
Twins	10	63	
Parity			0.628
1–4	29	733	
More than 4	13	239	
Never gave birth	17	363	

AVD = Assisted vaginal delivery; SVD = Spontaneous vaginal delivery

### Perinatal mortality and baby characteristics

Findings indicated of the neonatal characteristics only sex was not statistically significant when related with perinatal mortality (*p* = 0.001, ≤ 0.05). This implies that Apgar score, birth weight, age birth, foetal heart and presentation have a statistical relationship the history of perinatal mortality (Table 4).

**Table 4:** Bivariate analysis between perinatal mortality and neonatal characteristics,2022

Variable	Perinatal Mortality		*P*-Value
	Yes	No	
Neonate characteristics			
Sex			0.371
Male	34	690	
Female	25	645	
Birth Weight (Grams)			0.000
1000–1499	13	6	
1500–2499	21	223	
2500–4000	25	1099	
>4000	0	7	
Age Birth Category			0.000
Pre-term birth	17	41	
Term birth	42	1289	
Post term birth	0	5	
Foetal heartbeat (Beats per minute: bpm)			0.000
Not perceived	41	3	
110–160	18	1311	
<110/ >160	0	21	
Fetal Presentation			0.002
Head	52	1285	
Malpresentation	7	50	
Apgar Score			
Apgar Score 1st Minute			0.000
0–4	52	22	
5–7	4	103	
8–10	3	1210	
Apgar Score 5th Minute			0.000
0–4	44	4	
5–7	11	60	
8–10	4	1271	
Apgar Score 10th Minute			0.000
0–4	46	0	
5–7	7	7	
8–10	6	1328	

### Multiple logistic regression analysis between perinatal mortality and postpartum women and neonates

We conducted a multiple logistic regression analysis to adjust the variables, between perinatal Mortality and postpartum women and neonates listed in Table 5. These included commune of residence of the mothers, Birth weight, Age birth category, Foetal presentation, Apgar score in the 1st, 5th and 10th minute of the babies, history of perinatal mortality and type of Pregnancy of the postpartum women. Findings showed that there is a statistically significant association between Birth Weight (*p* = 0.004, ≤ 0.05), Apgar score of the 10th minute (*p* = 0.000, < 0.05) of the babies and Perinatal mortality. The odds ratio of Birth weight [AoR: 0.082, CI = 0.014–0.449, *p* ≤ 0.05) and Apgar Score in the 10th minute [AoR: 0.002, CI = 0.000- 0.043, *p* ≤ 0.05) indicates a significant low risk of Perinatal mortality.

**Table 5:** Multivariate analysis for perinatal mortality between postpartum women and neonatal characteristics,2022

Factors	Odds Ratio	95% Confidence Interval		*P*-value
		Lower	Upper	
Commune of Residence	1.300	0.557	3.035	0.543
Birth Weight	0.082	0.014	0.449	0.004^*^
Age Birth Category	0.390	0.041	3.682	0.412
Fetal Presentation	0.347	0.004	30.239	0.643
Fetal Heartbeats	7.102	0.315	159.754	0.217
Apgar score 1st minute	0.259	0.059	1.132	0.073
Apgar score 5th minute	1.562	0.129	18.899	0.726
Apgar score 10th minute	0.002	0.000	0.043	0.000
History of Perinatal Mortality	7.257	0.637	82.641	0.110
Type of Pregnancy	0.815	0.066	9.938	0.873

## Discussion

Studies indicate perinatal mortality occur during the first seven days of life, and one million newborns are estimated to die within 24 h of life [20]. In this study, PMR of 42.3 deaths per 1000 live births occurring at Beni GRH is slightly higher than the National PMR of 40 deaths per 1000 live births [6]. Similarly, 32 deaths per 1000 live births in Dr. RAU & CIRIRI hospital, Bukavu DRC and 27 deaths per 1000 live births in Lubumbashi DRC [12, 13]. Additionally, 34.7 deaths per 1000 live births in Ethiopia [4] and 38 deaths per 1000 live births in Uganda [21] and 5.0 per 1000 in Netherlands [22]. Interestingly, perinatal mortality risk in Netherlands is higher than in other European countries. The high PMR in DRC could be due to lack of initiatives to improve perinatal health inequalities like countries with least perinatal risk including national audit programme, antenatal surveillance and monitoring, and community-based interventions which could positively change the outcomes [22, 23]. In a study done by Mizerero, et al. reported lower utilization and inadequacies of unmet need of services including emergency obstetric and natal care seems to be systemic in nature reflecting low funding in the health sector [24]. DRC’s health system is laden by insecurity and decades of war resulting in meagre health budget at 10.3% in 2022. This falls short of meeting the Abuja Target of spending 15% of the national budget. Eventually, pressing health challenges. However, previous literature indicates less than 235 deaths per 1000 live births in Lomami hospitals of DRC [12].

The disparities may reflect different socio-economic status affecting maternal and neonates’ health, poor health system as well as contextual factors and time periods of various regions and countries which limit access to quality healthcare. Based on study findings, Apgar scores of less than 5 at 10 min and a birth weight of 1500 to 4000 g clearly confer an increased risk of perinatal death. This finding was consistent with previous studies [1, 17]. The likelihood of perinatal mortality to occur among babies with Apgar scores 0–4 in 10th minute is less likely than in 5th and 1st minutes. This is in line with a similar study [25, 26]. Studies indicate 10-point Apgar score is antiquated because of technological advances such as base excess (BE), blood pH, umbilical cord arterial lactate, and other metabolic acidosis indicators over the past 60 years since it was devised by Dr Virginia Apgar, 1952 [26, 27]. Nonetheless, studies still show Apgar score is suitable to assess clinical status and prognosis of new-born child. However, these advanced indicators are not available at Beni GRH. Thus, Apgar score has been used clinically to determine neonatal resuscitation. To improve precision, the Apgar score should be assessed given that it has some subjectivity. Furthermore, being born with LBW significantly is associated with increased perinatal mortality and is highlighted by the difference in birth weight-specific mortality. Thus, specific birth weight assists in prediction for the survival of neonates and decision-making for medics and parents.

However, previous literature [25–27] most pointed out sepsis as the common cause of perinatal death. The discrepancy between the current study findings and other study reports may be due to differences in the demography of the study population, the health care system and perhaps, more importantly, the methodology used for assigning a cause of death.

This study has some strengths and limitations. The strength of this study, it was a retrospective hospital-based with a high response rate (100%) and data was pooled together to create a large sample size. This enabled to identify significant factors of perinatal deaths to inform policy implementation. Finally, this study linked individual mothers with the facility they have been using and with the facility proximal to their communities. Most studies have mainly conducted surveys such as Demographic Health Surveys to ecologically link household and facility data; yet these studies introduce limitations such as linking individual mothers to facility that they did not use. Given that, Beni GRH was purposively selected, our findings and analyses cannot be generalized to the whole Province. Also, unconventional based deliveries, where healthcare quality is probable to be poor was not included. Despite the li-mitations however, it is strongly believed that this study provides valuable information in the field of this study.

## Conclusion

This study finding indicates perinatal mortality occurring at Beni GRH is a significant problem with 42.3 deaths per 1000 live births. The low Apgar score and birth weight were statistically significant determinants of perinatal mortality. Thus, interventions focusing on women education by increasing knowledge of key danger signs, improving emergency obstetric care, and neonatal resuscitation are proposed as a fast-track panacea to the preventable tragedy of perinatal mortality. Nonetheless, DRC must increase its efforts to comply with World Health Organisation ANC and PNC guidelines.

## Data Availability

Data is provided upon reasonable request.

## Abbreviations

ANC: Antenatal care
AOR: Adjusted Odd Ratio
BMI: Body mass index
CI: Confidence Interval
COR: Crude Odd Ratio
DRC: Democratic Republic of Congo
LBW: Low birth weight
NGO: Non-Governmental organization
MSF: Médecins Sans Frontières
SPSS: Software package for social sciences
GRH: General referral Hospital
PNC: Postnatal Care
SVD: Spontaneous Vaginal Delivery
WHO: World Health Organization
